# Refractory fungal infection: Three case reports highlighting good practice

**DOI:** 10.1016/j.mmcr.2024.100688

**Published:** 2024-12-04

**Authors:** Rosemary Barnes, David A. Enoch, Wendy Ingram, Jessica Martin, Jennifer Clay, Netta Tyler, P Lewis White

**Affiliations:** a Cardiff University School of Medicine, Heath Park Way, CF14 4YS, Cardiff, United Kingdom; b Cambridge University Hospitals NHS Foundation Trust, Hills Road, CB2 0QQ, Cambridge, United Kingdom; c University Hospital of Wales, Heath Park Way, CF14 4XW, Cardiff, United Kingdom; d Leeds General Infirmary, Great George Street, LS1 3EX, Leeds, United Kingdom; e St James Hospital, Beckett Street, LS9 7TF, Leeds, United Kingdom; f Public Health Wales Mycology Reference Laboratory, University Hospital of Wales, Heath Park Way, CF14 4XW, Cardiff, United Kingdom; g Centre for Trials Research/Division of Infection and Immunity, Heath Park, CF14 4YS, Cardiff, United Kingdom

**Keywords:** Refractory fungal infection, Breakthrough fungal infection, Invasive fungal disease, Antifungal resistance, Antifungal susceptibility testing, Fungal biomarkers, Antifungal stewardship

## Abstract

Refractory invasive fungal disease is a significant clinical problem, with high morbidity, mortality and costs. The complex causes of refractory infection include breakthrough infection due to antifungal resistance (both innate and acquired), suboptimal therapy and impaired immune responses in critically ill or immunocompromised patients. This case series details three reports on the identification and management of refractory fungal infections, two cases of azole resistance and one case of resistant candidiasis, highlighting the importance of accurate diagnosis, monitoring, implementation of biomarkers (serological markers, PCR), antifungal susceptibility testing and antifungal stewardship to optimise management and minimise risks of emergence of drug resistance.

## 1 Introduction

The treatment of breakthrough and refractory invasive fungal diseases (IFDs) represents a difficult clinical problem, potentially caused by multiple factors. Breakthrough infection can be defined as any fungal infection occurring while on antifungal therapy, including, but not limited to, infection due to resistant organisms [1]. Whereas, refractory infections are defined as a fungal infection with worsening signs or symptoms while on treatment [1]. Compliance and inadequate therapeutic drug levels are important considerations requiring therapeutic drug monitoring (TDM), particularly for triazole drugs. The role of the host immune response in predisposing an individual to IFD should not be underestimated.

Antifungal resistance may be innate or acquired. Ineffective binding to drug targets and efflux activities confer innate resistance across some species. *Aspergillus* spp., *Candida krusei* and most *Candida auris* isolates demonstrate innate resistance to fluconazole, and many other moulds are resistant to azoles, including Mucoromycota, and *Lomentospora* spp., with *Fusarium* spp. demonstrating variable susceptibility to azoles [2,3]. The emergence of acquired resistance from the selective pressures exerted by an antifungal agent may emerge in the host and the environment [2]. Mutations occur that confer resistance, including point mutations, gene duplications and transposon insertions, leading to the acquisition of resistance mechanisms [2]. Mutations leading to a growth advantage in higher concentrations of an antifungal drug result in antifungal tolerance, driven by genetic, physiological or epigenetic adaptations [2]. Azole resistance in *C. albicans*, for example, has developed because of prolonged fluconazole therapy for oral candidiasis in patients infected with HIV [2]. Aneuploidy (leading to gene duplication) and hypermutation with overexpression of drug targets and efflux pumps has been seen in *Cryptococcus neoformans* in response to azole therapy. Upregulation of specific genes such as *ERG5*, *ERG6* and *ERG25* can confer resistance to polyenes in *C. albicans* [2]. Environmental triazole resistance in *A. fumigatus* can emerge following exposure to fungicides used on crops, where insertion of tandem-repeats leads to increased expression of genetic point mutations of the *CYP51A* gene, reducing the binding of triazole therapy and impacting the ergosterol synthesis pathways; an increasingly common problem in clinical infection and associated with excess mortality [2,4,5]. Resistance can arise from phenotypic heterogeneity, such as biofilm formation, which may influence antifungal susceptibility [2].

Here we present three cases demonstrating some of the difficulties faced when managing such infections and emphasise the need for accurate diagnosis, susceptibility data (whether by conventional or molecular methods), TDM, and the distinction between clinical and laboratory resistance.

## 2 Case presentation

### 2.1 Case one: azole resistance in a patient with aplastic anaemia and recurrent IFD

A 21-year-old woman with a history of paroxysmal nocturnal haemoglobinuria presented with a second relapse of aplastic anaemia 7.5 years after initial diagnosis, while receiving ciclosporin. Conditioning (day 0) was initiated for an allogeneic stem cell transplant (SCT), during which the patient had a period of prolonged neutropenia (absolute neutrophil count (ANC) < 0.5 × 10^9^/L).

On Day 0, computed tomography (CT) of the thorax identified a 1.3 cm lesion with halo, typical of IFD, despite consistently negative serum *Aspergillus* polymerase chain reaction (PCR) [6] and galactomannan (GM, (Aspergillus antigen ELISA, Bio-Rad, UK) ) throughout this period and several weeks prior. Treatment with oral voriconazole 200 mg twice daily was initiated, but after 11 days, CT-sinuses showed septal deviation, mucosal thickening and opacification. Fear of refractory disease or potential mucormycosis lead to a switch to liposomal amphotericin B (L-AmB) 3 mg/kg daily, but subsequent renal toxicity and an allergic skin reaction led to a switch after 11 days (Day 22) to intravenous (IV) isavuconazole 200 mg daily. The patient was discharged 11 days later (Day 33) following graft rejection on oral isavuconazole, and then switched to outpatient care with posaconazole as secondary prophylaxis. They remained consistently leucocytopenic (0.1–0.2 × 10^9^/L) over the next 4 weeks, with negative serum *Aspergillus* PCR and GM.

On Day 73, following positive serum *Aspergillus* PCR and GM, and a large new lesion on CT-chest while receiving secondary prophylaxis, the patient started combination treatment (IV voriconazole 200 mg twice daily and anidulafungin 200 mg loading dose and 100 mg once daily). PCR and GM were positive (index: 1.1) on bronchoalveolar lavage fluid (Day 77). Initial TDM showed low voriconazole levels (0.73–0.77 mg/L) but satisfactory levels were achieved (Day 88). The patient remained on voriconazole before switching to oral isavuconazole 200 mg daily plus continued anidulafungin while undergoing conditioning for a second transplant (Day 93). Serum *Aspergillus* PCR remained positive for a further 2 weeks, while serum GM declined but did not become negative for a further 9 weeks. Anidulafungin was stopped and oral isavuconazole continued when the patient was discharged (on Day 126). Follow-up CT-chest imaging demonstrated radiologic improvement, with some persisting halos (Day 168). All biomarkers for *Aspergillus* remained negative for 4 months while on isavuconazole, with PCR positivity in serum documented on two occasions between Day 168 and Day 325.

On Day 325, the patient was readmitted with haemoptysis following a period of prolonged neutropenia/lymphocytopenia. Serum *Aspergillus* PCR was positive on consecutive occasions, with a negative GM index. CT-chest on Day 326 confirmed consolidation in the right middle and lower lobes. Bronchoalveolar lavage fluid obtained 1 week later (Day 332) demonstrated fungal hyphae on microscopy, with strongly positive GM and *Aspergillus fumigatus* identified by PCR possessing the TR_34_/L98H mutation (associated with azole resistance); no isolate was available for susceptibility testing. Given the detection of the TR_34_/L98H mutation and the development of refractory disease while the patient was on isavuconazole, patient intolerance to L-AmB, and previous best response observed while receiving an echinocandin, the patient was treated with caspofungin 70 mg loading dose/50 mg per day for 6 weeks.

Serum biomarkers became negative by Day 355. Repeat imaging on Day 397 and Day 425 were stable, with no evidence of cavitation, despite neutrophils remaining low. Following improvement in the neutrophil count to >1.0 × 10^9^/L on Day 545 after full donor engraftment, the patient has remained very well with no clinical signs of infection in the absence of secondary antifungal prophylaxis.

### 2.2 Case two: chronic disseminated candidiasis (CDC) in a patient with acute myeloid leukaemia

A 43-year-old man with acute myeloid leukaemia (AML) was referred for investigation of fever of unknown origin and consideration for SCT. At the time of referral, the patient was in morphologic and cytogenetic remission from AML following two cycles of chemotherapy.

Microbiology from the referring hospital was positive for *Candida albicans* (susceptible to fluconazole, amphotericin B and echinocandins) isolated from a single blood culture taken from a Hickman line 8 days after AML was diagnosed (Day 0). A sample was not available for genotyping. The patient was treated with IV fluconazole (400 mg once daily after an 800 mg loading dose) for 2 days, then switched to L-AmB 3 mg/kg due to hepatotoxicity. L-AmB treatment continued for 2 weeks post negative blood culture. However, despite returning five sets of negative blood cultures following L-AmB treatment, further antifungal treatment with caspofungin 70 mg IV once daily for 4 weeks, and empiric prednisolone for suspected haemophagocytic lymphohistiocytosis, fever persisted.

**Fig. 1:**
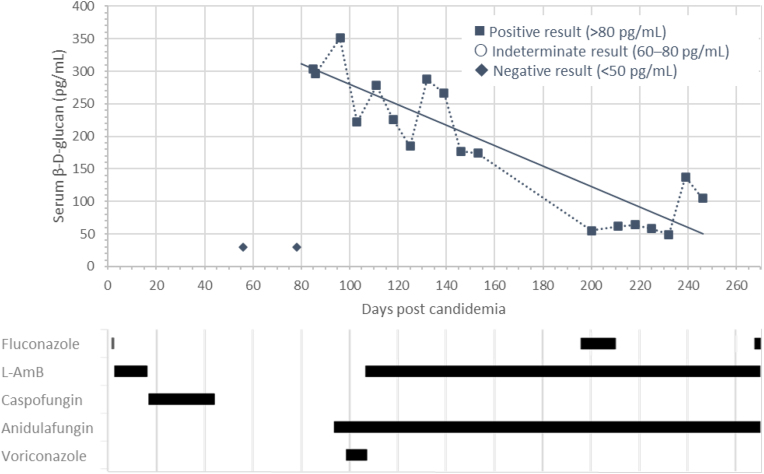
Case two: Serial serum β-D-glucan levels in a patient with AML and antifungal treatments administered. Linear trend line for positive values only. AML, acute myeloid leukaemia; L-AmB, liposomal amphotericin B.

**Fig. 2:**
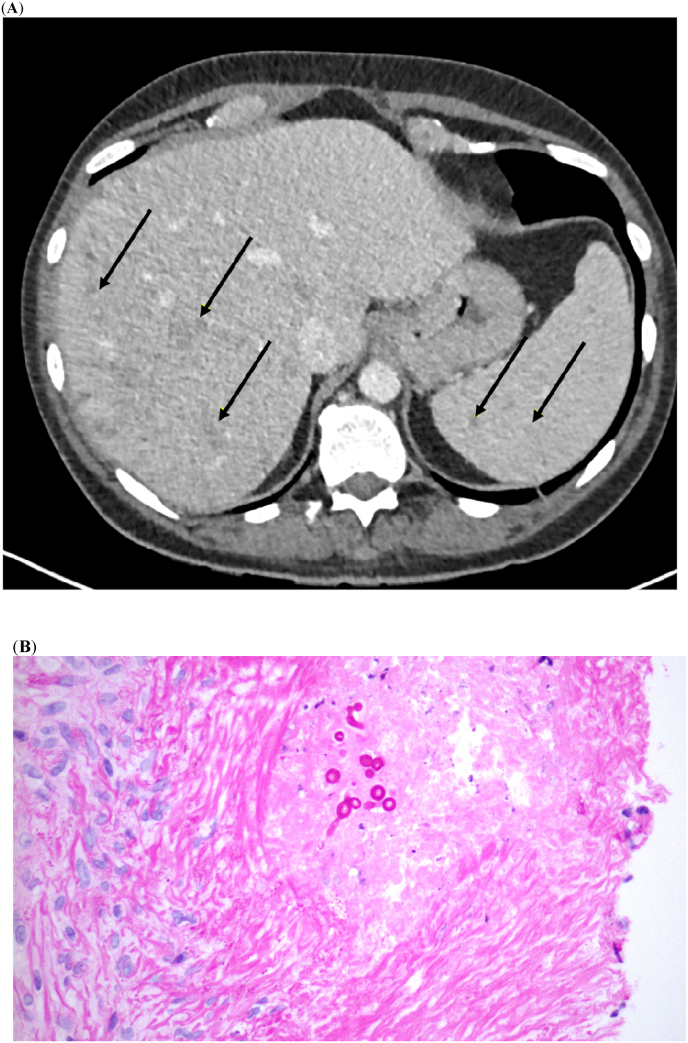
Case two: (**A**) axial image from a portal phase CT demonstrating (arrows) numerous hypo-attenuating lesions in the liver and spleen, (**B**) liver biopsy with parenchyma showing a single large focus of coagulative necrosis with palisaded epithelioid histiocytes, fibrosis, lymphoplasmacytic infiltrate and a few fungal cells with short hyphae.

On admission (Day 85 post-candidemia), the patient had mild hepatomegaly (2 cm), low haemoglobin (Hb) levels (78 g/L), and elevated neutrophil (16.2 × 10^9^/L) and platelet (8.62 × 10^10^/L) counts. Biochemistry results showed hypoalbuminaemia (19 g/L), and elevated alkaline phosphatase (422 U/L) and alanine transaminase (77 U/L). Serum β-D-glucan was also noted to be positive (Fig. 1). Echocardiography was unremarkable. CT imaging showed heterogeneous uptake in the liver and spleen, with increased multiple small foci of activity compared with a prior scan, particularly in the spleen (Fig. 2A). A liver biopsy performed on Day 97 showed hyphal forms as typically developed by yeasts, (Fig. 2B), but cultures and pan-fungal PCR (performed at the national mycology reference laboratory) were negative.

Treatment was initiated with anidulafungin 100 mg IV once daily (after a loading dose of 200 mg IV) on Day 94, 3 days before the biopsy was taken, with oral voriconazole (400 mg twice daily loading dose followed by 200 mg twice daily thereafter) added on Day 99. After no clinical response, the patient discontinued voriconazole and switched to L-AmB 3 mg/kg on Day 107. Repeat CT imaging showed persistence of lesions on Day 143. The biopsy may have been culture negative due to anidulafungin being commenced prior to the biopsy, whilst panfungal PCR is recognised to be less sensitive than organism-specific molecular assays. Nonetheless, the imaging and liver biopsy support the chronic disseminated candidiasis diagnosis. The EORTC-MSG criteria [7] were used to classify invasive fungal disease.

The patient was discharged to the referring hospital on Day 148 on dual antifungal therapy and readmitted on Day 191 for elective SCT. Fluconazole 400 mg IV once daily was added to the dual antifungal regimen on Day 196 but was stopped on Day 210 after no benefit was seen and the patient remained febrile. All cultures were negative. On Day 216, the patient received his BMT and engrafted successfully. The patient developed a rash that was confirmed as acute graft-versus-host disease (GvHD) on Day 230, which settled with prednisolone. The pyrexia subsided and they were discharged on Day 254 when their white blood cell count was 2.6 × 10^9^/L and neutrophil count was 2.33 × 10^9^/L, and they recommenced fluconazole on Day 268. An abdominal CT scan on Day 273 showed resolution of the lesions.

### 2.3 Case three: azole-resistant pulmonary aspergillosis in a patient with relapsed Hodgkin lymphoma

A 45-year-old male on salvage gemcitabine chemotherapy for relapsed Hodgkin lymphoma (HL) post-allogeneic SCT was admitted following a 2-week history of progressive tiredness, shortness of breath, fever and neutropenia (Day −4).

On admission, the patient was taking prophylactic oral itraconazole 120 mg twice daily and oral co-trimoxazole 480 mg once daily, and a chest x-ray showed diffuse bilateral lower zone opacification. Initial tests for infection were negative. No improvement was seen with empiric antibacterial treatment for neutropenic chest infection (IV piperacillin-tazobactam 4.5 g four times daily plus oral clarithromycin 500 mg twice daily). On Day −1, itraconazole prophylaxis was switched to L-AmB 3 mg/kg, pending further results from high-resolution CT (HRCT). Symptoms included a progressive cough with light haemoptysis, bilateral basal crepitations, persistent fever and 92 % oxygen saturation on air.

Sputum cultures grew *Aspergillus fumigatus* (Day 0). The clinical picture was complicated by a vasculitic rash on both feet. Later diagnostic tests for vasculitis were negative. On Day 4, the patient's symptoms improved, and antifungal therapy was switched to voriconazole (IV loading dose followed by oral switch). Susceptibility results were not available at this time. By Day 7, the patient had deteriorated, with rising C-reactive protein, reduced oxygen saturation, and worsening fever. They were switched back to IV L-AmB 3 mg/kg and improved clinically by Day 11. *Aspergillus fumigatus* sensitivities returned on Day 20 and revealed resistance to itraconazole (Table 1).

**Table 1:** Case three: Culture and sensitivity results (Clinical & Laboratory Standards Institute (CLSI) methodology) of *Aspergillus fumigatus* isolated from the sputum of a patient with relapsed HL.

Organism	Agent	MIC (mg/L)	Interpretation
*Aspergillus fumigatus*	Itraconazole	8.0	Resistant
	Voriconazole	2.0	Intermediate
	Posaconazole	1.0	Intermediate
	Isavuconazole	2.0	Intermediate
	L-AmB	≤2.0	Sensitive
	Caspofungin	≤0.5	Sensitive

HL, Hodgkin lymphoma; L-AmB, liposomal amphotericin B; MIC, minimum inhibitory concentration.

The patient was discharged on outpatient antimicrobial treatment with L-AmB 3 mg/kg daily. Treatment was completed on Day 61, with good clinical and radiologic response and no adverse effects. Despite sensitivity concerns (Table 1), the patient was switched to posaconazole 300 mg once daily as secondary prophylaxis, with monthly serological monitoring (*Aspergillus* antigen (GM) and beta-D glucan) and TDM. No recurrent *Aspergillus fumigatus* infection occurred during 2 years of follow-up while on posaconazole as secondary prophylaxis, despite the patient experiencing COVID-19, complex infection and haematologic issues in this period.

## 3 Discussion

The first case describes a patient with invasive pulmonary aspergillosis probably resulting from fungal sinusitis. Interestingly, biomarkers were negative during the early stages of infection. This has been described in the literature before in cases of sinusitis [8], and we hypothesize that this could be because fungal serologic markers (GM, β-D-glucan) and fungal DNA may not be released into the bloodstream until extensive breakdown of tissue barriers has occurred. However, a solitary lung nodule was noted on admission, so systemic infection with low fungal load or another infection such as mucormycosis could have been present at Day 0. Infection progressed on azole therapy, albeit with inadequate therapeutic drug levels at times. The patient was intolerant of L-AmB.

Subsequent biomarkers and bronchoalveolar lavage identified *Aspergillus* spp*.,* and although cultures were negative, the TR_34_/L98H mutation associated with azole resistance in *Aspergillus fumigatus* was identified by molecular methods [9]. This demonstrates the value of non-culture-based assays for the detection of resistance, such as *A. fumigatus* to azoles, allowing the identification of resistance directly from samples and overcoming the limitations of other sampling methods. The patient responded to echinocandin and azole combination therapy in accordance with international guidelines [10]. L-AmB is also recommended, but the clinicians felt it was contraindicated due to presumed intolerance.

This case highlights the problems of azole resistance, probably acquired from the environment, (although the patient received prolonged periods of azole therapy with sub-therapeutic levels) the importance of TDM [11], the utility of non-culture diagnostics, and compliance with guidelines.

Case two describes a case of proven fungal infection (candidemia) leading to likely CDC in a patient with AML. CDC represents a refractory infection typically manifesting after neutrophil recovery following immune suppression, with granulomatous lesions throughout the reticular endothelial system [12,13]. Serum β-D-glucan was positive on admission and persisted throughout the course of infection, albeit with a slow downward trend. Although clearance of serum β-D-glucan is known to be slow and a downward trend is considered favourable, it is possible that slow clearance was indicative of refractory disease. Biomarkers can be considered a useful tool in monitoring response to treatment and resolution of infections, although the slow clearance of β-D-glucans limits its value here. The fungal burden is characteristically low and positive identification of the organism/infection can be difficult.

CDC results from immune dysfunction and there is debate as to whether this is an immune reconstitution inflammatory syndrome (IRIS) [14] or immune paresis [15]. While fever and inflammatory markers often respond to corticosteroid treatment [16], the resolution of disseminated fungal lesions may require other immunomodulating agents in addition to prolonged antifungal therapy [17]. Case two highlights the importance of the host immune system in determining recovery. This difficult-to-treat prolonged infection, rare now in the era of fluconazole prophylaxis, emphasizes that antifungal agents are not the sole determinants of patient outcomes. Immunophenotyping and cytokine profiling may identify defects in innate immune pathways that could guide management [14]. Adjuvant therapy is often required, but relapse of underlying disease is strongly predictive of mortality, so delays to further chemotherapy or transplantation should be avoided if possible [18].

Case three describes a man with relapsed HL and GvHD following SCT treated with gemcitabine who developed a fungal pulmonary infection while on itraconazole prophylaxis and steroid treatment. Gemcitabine inhibits DNA synthesis in neoplastic cells and, while a cause of marrow suppression and lymphopenia, is not a major cause of neutropenia unless used in combination with other chemotherapy [19]. Sputum culture yielded *Aspergillus* spp., leading to a switch to voriconazole and a further switch 3 days later to L-AmB due to lack of clinical response and previous use of azole prophylaxis, resulting in a resolution of the infection symptoms. Subsequent susceptibility results showed resistance to itraconazole and intermediate susceptibility to other triazoles. The case demonstrates the value of AFST testing in informing treatment decision-making and the need in this case to alter patient management. This patient may have benefited from non-culture-based sensitivity testing, as demonstrated in Case 1. This case may represent a breakthrough infection on itraconazole, which the patient had been on for nearly 10 months prior to this episode. TDM does not appear to have been undertaken during this period, but it is possible that azole resistance developed due to prolonged therapy [20]. The brief duration of voriconazole treatment is insufficient to make any judgement concerning treatment failure, but concerns are justified given infection progressed despite azole prophylaxis. Post successful L-Amb treatment, the patient remains well on secondary prophylaxis with posaconazole and continued TDM. No CT imaging was performed, so the extent of disease is unknown, but it is possible that this represents a case of chronic pulmonary aspergillosis following SCT with GvHD. The case highlights risk factors that persist past the initial post-transplant phase.

This case demonstrates that not all invasive fungal diseases are associated with profound neutropenia. Many cases occur late after transplantation and are often associated with GvHD, steroids, and defective cell-mediated immunity [21,22], leading to a more chronic form of disease. It also shows the usefulness of susceptibility testing results in guiding patient management and emphasizes the “90:60 rule”: susceptible isolates respond to therapy approximately 90 % of the time, while resistant isolates respond approximately 60 % of the time [23].

In conclusion, these cases demonstrate the difficulties with the management of refractory fungal disease and the growing problem of azole-resistant fungi. They highlight the need for diagnostic and clinical monitoring and the importance of antifungal stewardship [24].

## CRediT authorship contribution statement

**Rosemary Barnes:** Writing – review & editing, Writing – original draft, Conceptualization. **David A. Enoch:** Writing – review & editing, Investigation. **Wendy Ingram:** Writing – review & editing, Investigation. **Jessica Martin:** Writing – review & editing, Investigation. **Jennifer Clay:** Writing – review & editing, Investigation. **Netta Tyler:** Writing – review & editing, Investigation. **P Lewis White:** Writing – review & editing, Investigation, Conceptualization.

## Consent

Written informed consent was obtained from the patients for publication of this case series and accompanying images. A copy of the written consent is available for review by the Editor-in-Chief of this journal on request.

## Funding

Gilead Sciences initiated and funded this publication. Although they had no input into the content, they performed an editorial review.

## Conflicts of interest

R.B. has no conflicts to declare. D.A.E. has no conflicts to declare. W.I. has no conflicts to declare. J.M. has received honoraria from Gilead Sciences, 10.13039/501100005612Shionogi and 10.13039/501100012276Tillotts Pharma. J.C. has received honoraria from Gilead Sciences, Jazz Pharma and Hartley Taylo. N.T. delivered an Anti-infective Masterclass on Antifungal Stewardship at 10.13039/501100002926Cambridge University Hospitals in June 2019 for 10.13039/100004319Pfizer and received funding from Gilead Sciences to attend 10.13039/100005564Gilead CARE Meeting in June 2022 in Berlin. P.L.W. performed diagnostic evaluations and received meeting sponsorship from Associates of Cape Cod, Bruker, Dynamiker, and Launch Diagnostics; speaker's fees, expert advice fees and meeting sponsorship from Gilead; speaker and expert advice fees from Pfizer; expert advice fees from F2G; and expert advice fees from 10.13039/100030679Mundipharma.
